# Agronomic Evaluation and Molecular Cytogenetic Characterization of *Triticum aestivum* × *Thinopyrum* spp. Derivative Breeding Lines Presenting Perennial Growth Habits

**DOI:** 10.3390/plants12183217

**Published:** 2023-09-09

**Authors:** Robin Morgan, Tatiana Danilova, Matthew Newell, Xiwen Cai, Stephen Jones

**Affiliations:** 1 WSU Breadlab, Department of Crop Science, Washington State University, 11768 Westar Ln, Burlington, WA 98233, USA; joness@wsu.edu; 2 Wheat, Sorghum & Forage Research Unit, USDA-ARS, Lincoln, NE 68583, USA; tatiana.danilova@usda.gov (T.D.); xiwen.cai@usda.gov (X.C.); 3 Cowra Agricultural Research Station, NSW Department of Primary Industries, 296 Binni Ck Rd, Cowra, NSW 2794, Australia; matt.newell@dpi.nsw.gov.au

**Keywords:** perennial wheat, wild relatives, cytogenetics, amphiploid, *Thinopyrum*

## Abstract

The transition from annual to perennial growth habits can contribute to increased sustainability and diversification of staple cropping systems like those based on annual wheat. Amphiploids between *Triticum aestivum* and *Thinopyrum* spp. can present a wheat-like morphology and post sexual cycle regrowth. The complex and unpredictable nature of the chromosomal rearrangements typical of inter-generic hybrids can hamper progress in the development of this new crop. By using fluorescence in situ hybridization, we described the genomic constitution of three perennial wheat breeding lines that regrew and completed a second year of production in field conditions in Washington state (USA). Two breeding lines presented stable, 56-chromosome partial amphiploids; however, their chromosome composition differed significantly. The third breeding line presented an unstable karyotype with a chromosome number ranging from 53 to 58 across eight individuals. The agronomic performance of the perennial breeding lines was evaluated for two growing seasons from 2020 to 2022. The grain yields of the perennial lines were lower than the grain production of the annual wheat control line in the first season. The perennial lines displayed vigorous regrowth after the initial harvest; however, worsening environmental conditions in the second season of growth hampered subsequent growth and grain yield. This information facilitates the breeding work necessary to improve key traits by grouping agronomically valuable individuals according to their genomic constitution.

## 1. Introduction

Traditional grain cropping systems have been threatened by chaotic climate trends [1] and by the depletion and disruption of soils [2]. In order to extend the longevity of such cropping systems, crops can be bred to overcome some of the negative externalities associated with their production, including higher rates of soil loss, changed landscape hydrology, declining soil organic carbon, and nutrient runoff [3,4]. The development of a perennial grain crop based on common wheat (*Triticum aestivum* L.) is an example of an ideotype that could address the need for improved crop sustainability and resilience in the face of climate variability.

Hybridization between annual *Triticum* crop species and perennial relatives provides an avenue to combine their polycarpic habits with grain quality in a new crop. This direction has been pursued by several researchers in the last hundred years [5,6,7,8,9]. There have been encouraging results in progressing towards a true perennial cereal through hybridization, although this has been largely dependent on the environment in which they are grown [8,10,11], and most lack a robust perennial habit and stability in grain yield. Breeding lines of hybrid perennial cereals have exhibited higher mineral and protein content than annual cultivars [12], with useful grain functionality across a range of products [13]. Perennial grains will need to be profitable if they are to be adopted more widely in agriculture and accepted into traditional markets [14]. Therefore, the development of a more productive germplasm is a priority. More recently, a stable *T. aestivum* and *Thinopyrum ponticum* (Podp.) Barkworth and D. R. Dewey amphiploid (×*Tritipyrum aaseae*) has demonstrated a polycarpic habit over two growing seasons and has progressed to commercial cultivar release as ‘Salish Blue’ [9]. This cultivar presents a wheat-like morphology and a growth habit defined as post-sexual cycle regrowth (PSCR). Flowering is largely indeterminate, with continuous production of tillers from the crown, allowing the plant to stay in reproductive mode rather than senescing. This demonstrates that developing cultivars with improved traits is possible.

Many species from the genus *Thinopyrum* A. Löve are capable of being hybridized with wheat [15,16]. The controlling genes for PSCR have been traced to chromosome 4E of *Thinopyrum elongatum* (Host) D.R. Dewey (2n = 2x = 14) [8]. In both monosomic and disomic additions and substitutions of chromosome 4E, PSCR was conferred in a wheat background. However, the growth habit was not as robust in these lines, as displayed in the full amphiploid progenitor. PSCR appears to be a polygenic trait and is not easily conferred by straightforward introgression from perennial to annual species [10,17]. Indeed, the screening of over 150 wheat × wheatgrass derivatives in a series of field experiments [18] found that lines capable of PSCR contained seven or more chromosome pairs from the perennial parent. Many fertile and stable wheat derived amphiploids have been formed at the octoploid level (2n = 56) across a range of *Thinopyrum* species [19,20]. *Thinopyrum* ssp. includes species and ecotypes with different ploidy levels starting from diploid (2n = 2x = 14) to decaploid (2n = 10x = 70). Polyploid *Thinopyrum* ssp. are allopolyploids [15,16,21], and their crosses with wheat can result in fertile partial amphiploids with random chromosome mixtures originating from different *Thinopyrum* sub-genomes [22]. This poses difficulty for any breeding program, as each time the primary partial amphiploid is produced, the synthetic genome may contain a different set of chromosomes [23,24,25,26]. Any interbreeding between these partial amphiploids could result in a loss of donor chromosomes, leading to instability and a loss of the perennial habit [27]. Despite this, many of the best performing perennial wheat lines have been derived from polyploid wheatgrass parents [10,28]. These represent an important source of germplasms for further development of perennial wheat. In order to allow for further improvement of the germplasm, agronomically valuable lines can be genetically characterized and divided into groups according to their genomic constitution [9].

Here, we report the use of fluorescence in situ hybridization (FISH) analysis to characterize the genomic constitution of three *T. aestivum* × *Thinopyrum* spp. breeding lines that successfully regrew in field conditions in Washington state, USA. Few previous studies have used FISH to quantify the genomic constitution of perennial cereals at the chromosome level. This work presents information on breeding lines for the development of a novel perennial grain staple crop.

## 2. Results

### 2.1. Agronomic Evaluation

**Figure 1:**
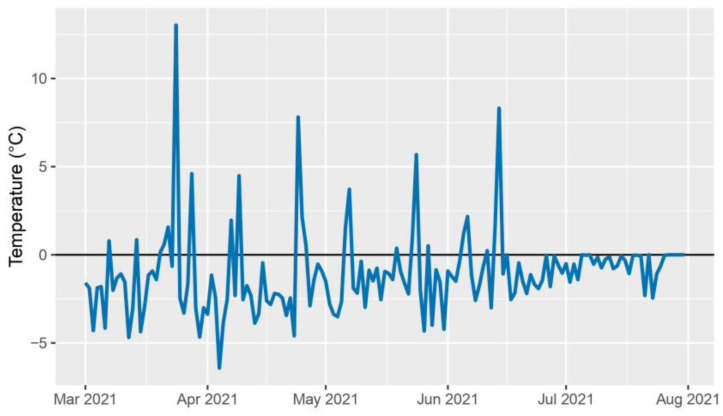
2021 daily average air temperature deviation from the ten-year daily average air temperature (2010–2020).

**Figure 2:**
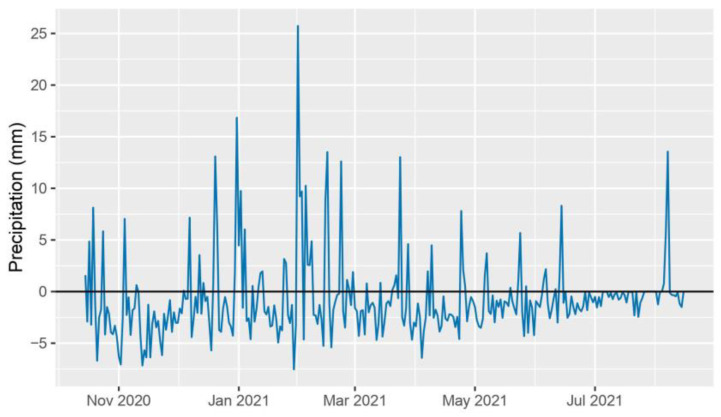
2021 daily average precipitation deviation from the ten-year average daily precipitation (2010–2020).

The 2020–2021 crop year was characterized by exceptionally hot and dry conditions. The deviation from the ten-year daily average peaked at the end of June and exceeded the long-term average by up to 10 °C (Figure 1). Rainfall also presented an anomalous trend, with the average monthly precipitation being 30% less than the long-term average over the growing season. The period preceding the high temperatures, from 1st March to 26th June 2021, overall presented a reduced level of rainfall when compared to the ten-year daily average precipitation, accumulating a rainfall deficiency of 140.1 mm (Figure 2). Perennial hybrids CS14PHR21, 202, 11955, and wheat control CS14PVT03 successfully completed the growth cycle spanning from October 2020 to August 2021.

Three weeks after the first-year harvest, CS14PHR21, 202, and 11955 clearly exhibited vigorous PSCR. The plant growth accurately replicated the original planting pattern, dismissing any attribution of the regrowth from volunteer seedlings arising from seed loss during harvesting operations. CS14PVT03 did not present any sign of PSCR, confirming its annual growth habit.

In September 2021, CS14PHR21, 202, and 11955 did not show any evident reduction in the original plant stand, with all three entries scoring close to 100% for regrowth (Table 1). In their second year of growth, CS14PHR21, 202, and 11955 did not present a renewal of the vernalization requirement. The plants continued advancing through the growth stages, such as tillering, stem elongation, heading, and flowering, independent of the environmental conditions (for example, decreasing air temperature). At this stage, CS14PHR21, 202, and 11955 had 80% of the plants at boot stage (BBCH 41–49), with the remaining 20% of plants initiating flowering (BBCH 51–59). Following mowing, the plants grew new leaf tissue per cut culm within 30 days (December 2021). From January to May 2022, the damage to the plants caused by water logging associated with severe rain events in Fall 2021 become progressively more evident in the form of visual gradients of plant necrosis. Weed pressure from *Trifolium pratense* L. negatively impacted the breeding lines’ plots. The breeding lines CS14PHR21 and 202 completed a second cycle of production in field conditions with one plot each, while all plots of 11955 succumbed to the excessively challenging conditions, despite presenting vigorous regrowth into the second year.

Observations of stripe rust (*Puccinia striiformis* Westend f. sp. *tritici*) infection suggested that CS14PHR21 and 202 were moderately resistant and 11955 was moderately susceptible to the disease throughout the two growing seasons (2020–2021, 2021–2022). (Table 2). Breeding line 202 displayed phenotypic segregation within the plant population and also displayed varied severity of stripe rust infection between plants. The severity of the disease infection was similar between the perennial line CS14PHR21 and the annual wheat line CS14PVT03; however, the disease severity was approximately 50% greater in perennial line 11955 compared to the other entries.

### 2.2. Kernel Characteristics

**Figure 3:**
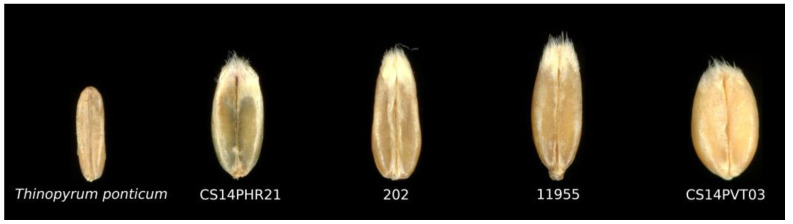
Comparison of seed shape and size between perennial hybrids CS14PHR21, 202, and 11955 compared to annual wheat CS14PVT03 and *Th. ponticum*.

The kernel characteristics varied between entries (Table 3). The thousand-kernel weight was similar between the perennial entries CS14PHR21 and 202, and 28% lower than the annual wheat control on average. Breeding line 11955 presented a thousand-kernel weight that was significantly higher than the other two perennial lines and significantly lower than the annual control; 18% on average. The kernel width of the annual control was, on average, 40% higher than the kernels of the perennial breeding lines. There was a similarity between the entries in terms of kernel length, although there was a clear indication that the kernels of entry 11955 were 10% longer on average than the other entries assessed (Figure 3).

### 2.3. Grain Yield

The average first-year grain yield ranged from 50% to 77% of the annual control for CS14PHR21 and 11955 respectively, while 202’s yield was 28% of the annual control (Table 4). The average yield of breeding line CS14PHR21 was significantly lower than the annual control but similar to 11955, which in turn was similar in grain yield to the annual wheat control. The second-year grain yields were less than half of the first-year results, with the highest being a single plot of line 202 at 282.33 Kg/ha, followed by a single plot of CS14PHR21 at 112.96 Kg/ha. Due to the low survival rate, the difference observed between the second-year grain yields of CS14PHR21 and 11955 was not statistically significant.

### 2.4. Cytological Analyses

#### 2.4.1. Wheat × *Thinopyrum* Amphiploids

**Figure 4:**
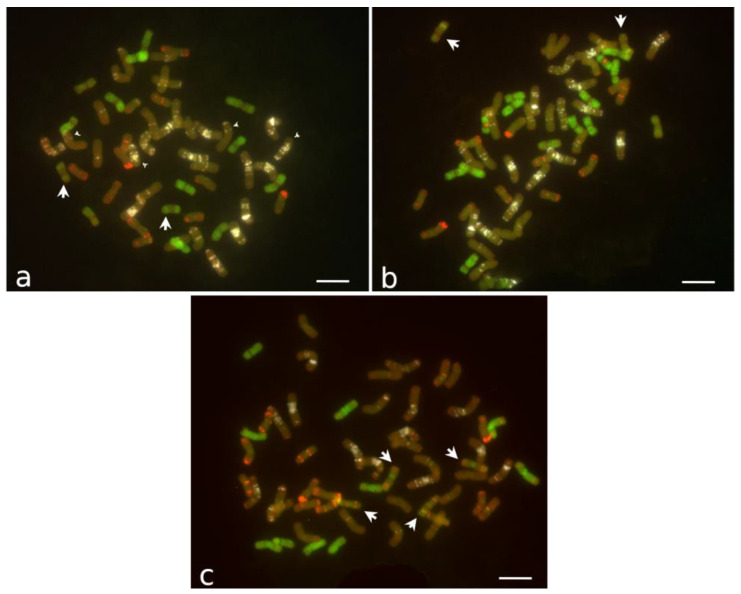
FISH of mitotic chromosomes. (**a**) Line CS14PHR21, 2n = 56, arrowheads pointing to reciprocal translocation chromosomes involving wheat 1A and 1B. (**b**) Line 11955, 2n = 56. (**c**) Line 202, plant with 2n = 55, 41 wheat (1D absent) and 14 *Thinopyrum* chromosomes. Arrows indicate *Thinopyrum* chromosomes partially painted with probe pTe. Probes pTe, (GAA)n, and pAs1 are shown in green, white, and red, respectively. Chromosomes counterstained with DAPI are shown in the dark orange pseudocolor. Bar represents 10 μm.

The chromosomal spreads of four individuals of CS14PHR21 were analyzed using FISH. All the plants had 2n = 4x = 56, including 14 *Thinopyrum* chromosomes and 42 wheat chromosomes (Figure 4a). Twelve alien chromosomes were painted with pTe probe along their entire length except the centromere area, but one pair had only the distal parts of the arms painted. In all the analyzed individuals, the pair of wheat satellite chromosome 1B had a shortened long arm, missing distal (GAA)n sites, which were substituted with chromatin with weak pAs1 signals. The normal chromosome 1A was not detected; instead, one pair of large metacentric chromosomes with interstitial and telomeric (GAA)n bands on one arm was found. Based on the distribution of (GAA)n and pAs1 signals on these two pairs of chromosomes, one can suggest a reciprocal 1AS–1BL translocation, with the distal end of 1AS bearing the pAs1 site translocated to 1BL and approximately half of 1BL translocated to 1AS.

Four individuals of line 11955 were analyzed, and three had 2n = 4x = 56, with genome composed of 16 *Thinopyrum* and 40 wheat chromosomes (Figure 4b). All the individuals missed chromosome pair 6A. Based on the chromosome morphology and FISH painting, this line differs from CS14PHR21 in the composition of the *Thinopyrum* chromosomes. A pair of chromosomes with a bright pAs1 signal at the centromeric area and a pair of chromosomes partially painted with the pTe probe and weak (GAA)n and pAs1 bands were present in 11955 but not observed in CS14PHR21. Conversely, a pair of partially pTe-painted chromosomes with no pAs1 and (GAA)n signals found in line CS14PHR21 were absent in breeding line 11955. One 11955 individual had 2n = 55 and differed from the other three based on the absence of a wheat B-subgenome chromosome.

Line 202 included plants with diverse karyotypes. Out of the seven individuals analyzed, two had 55, three had 56, one had 57, and one had 58 chromosomes. The numbers of *Thinopyrum* chromosomes varied from 13 to 16. Two plants had 41 wheat chromosomes with one chromosome 1D or one chromosome 3D absent. The number of *Thinopyrum* chromosomes completely painted with the pTe probe varied from 10 to 13. In all the plants, two types of chromosomes partially painted with the pTe probe with specific (GAA)n and pAs1 bands (designated F and K) presented in different combinations. In one plant, they were present as two pairs (2F + 2K), (Figure 4c); in three plants, they were present as one of each (F + K); one plant had 2K; another plant had telo F + 2K; and the last plant had a translocation chromosome with only one arm painted with pTe probe.

#### 2.4.2. *Thinopyrum Accessions*

To interpret the chromosome composition of wheat × *Thinopyrum* amphiploids, we performed a FISH analysis of potential parental *Thinopyrum* species: *Th. ponticum* accession PI 547312 and *Th. elongatum* accessions PI 206624 and W6 21870. All the plants were decaploids, but some variation in the chromosome numbers was observed. Five plants of PI 206624, two plants of W6 21870, and one plant of PI 547312 had 2n = 10x = 70. One plant of W6 21870 had 69 chromosomes, two plants of PI 547312 had 71 chromosomes, and one plant had 73 chromosomes.

The combination of probes used for the FISH analysis of wheat × *Thinopyrum* amphiploids was applied to the three *Thinopyrum* accessions (Figure S1). FISH painting revealed that all three accessions had subsets of chromosomes differing in the distribution of the pTe probe, originated from a diploid *Th. elongatum*. On most chromosomes, pTe hybridized near equally along their entire length except the centromeric area, and on a subset ranging from 10 to 14 chromosomes, pTe hybridized only to the distal ends of the chromosomes. Within each accession, the plants showed diversity in their distribution of pAs1 and (GAA)n repeats.

## 3. Discussion

In the first growing season, CS14PHR21, 202, and 11955 presented a winter wheat phenology, as clearly shown by their vernalization requirement. However, between flowering and mid-seed development (BBCH 61–77), these breeding lines initiated a new phase of tiller development. The primary set of tillers entering the stem elongation phase appeared to not be suppressing the activity of the axillary meristems, allowing for a continuous production of biomass. PSCR has been described as an additional phase of biomass production following the completion of the reproductive cycle [8]. In the breeding lines presenting PSCR, senescence is compartmentalized to individual reproductive tillers rather than being a whole plant phenomenon as observed in annual wheat. After mechanical harvesting removed most of the above-ground biomass, the plants resumed growing, morphologically resembling the leaf development stage (BBCH 11–15). The removal of any hormonal control from the reproductive tillers, paired with a fully developed and established root system, stimulated a rapid regrowth of the canopy.

The plants, after a vegetative phase lasting three months (September to November), transitioned to reproduction as these vegetative tillers began to mature. This indicates that the vernalization requirement met during their first growing season remained valid, even for the second phase of growth. The growth habit presented by the genotypes is a hybrid between the wheat and wheatgrass parents. The plants showed perennial growth habits like the *Thinopyrum* spp. parent, but lost the winter dormancy strategy typically observed in *Thinopyrum* spp., behaving like a winter wheat plant that resumed growth after the cold season. The plant growth was modulated only by the air temperature’s effect on biomass production rather than by having to overcome a physiological requirement for cold to transition from the vegetative to the reproductive phase. The plants, with their growth stages decoupled from seasonality, invested in a novel reproductive effort by elongating their tillers and progressing towards flowering for a second time in less than six months since the first grain harvest. However, these efforts were doomed to fail because of the low temperatures that minimized their chances for successful fertilization and seed set [29]. Wheat plants are known to be more susceptible to frost damage in their reproductive phase compared to during their vegetative growth [30]. Mowing can prevent the expenditure of the plant’s resources, increasing the likelihood of its survival through a second winter in field conditions [31]. A comparable result with additional agro-ecological benefits could be achieved by integrating a perennial grain crop with grazing animals [32,33]. The removal of the photosynthetic plant tissues, together with the reduced growth rate determined by a low air temperature, allows for these non-dormant plants to present a morphology more congruent with the time of year. This management technique delays the reproductive phase, allowing heading and flowering to occur in late Spring or early Summer. Mowing/grazing schedules would need to be varied according to weather patterns, in particular the air temperature. In Western Washington State environments, with grain production the main focus, the plants would be mowed one or two times during their second year of growth. The first defoliation would take place between two and three months after harvest, with a subsequent cut approximately six months after harvest. The observed first- and second-year grain yield was in accordance to values reported in the literature [34]. The second-year grain yield was lower than the first, Likely as a consequence of the waterlogging damage.

*T. aestivum* and *Thinopyrum* spp. have many contrasting traits, and one of the most pertinent traits for grain production is seed size. The domestication of common wheat favored a larger endosperm; a target still adopted in contemporary wheat breeding programs [35]. The inclusion of *Thinopyrum* spp. chromosomes has the potential to affect the seed phenotype in terms of its size and shape. Breeding lines 202, CS14PHR21, and 11955 produce a wheat-like seed, characterized by a more slender shape due to a smaller width, a greater length, and a smaller thousand-kernel weight than the annual winter wheat control (Table 3). *T. aestivum* × *Thinopyrum* spp. amphiploids have been reported to present a higher whole-grain protein content [36] and a lower flour yield [12] than annual common wheat. This indicates that the addition of *Thinopyrum* spp. chromosomes alters the nutritional composition of the kernels and increases valuable compounds ranging from proteins to minerals like Ca, Fe, Zn, and P [12]. Epidemiological research [37] has indicated a lack of whole grains as a prominent dietary risk factor for the deterioration of human health. *T. aestivum* × *Thinopyrum* spp. amphiploids present a novel ideotype capable of addressing these issues.

The diversity in agronomic performance among the analyzed partial amphiploids reflects the variability of their chromosome constitution. The breeding line CS14PHR21 contains a *Thinopyrum* genome complement composed of 14 chromosomes, with a few weak pAs1 or (GAA)n signals and a complete common wheat genome (Figure 4b). However, it appears that a structural chromosomal rearrangement, such as a reciprocal translocation, may have taken place between wheat chromosome 1A and 1B. This hypothesis is supported by the chromosome morphology and distribution of pAs1 and (GAA)n repeats on the two chromosome pairs.

Line 11955 has 40 wheat chromosomes and 16 *Thinopyrum* chromosomes, with at least four pairs different from those observed in CS14PHR21. Line 11955 lacks chromosome pair 6A, but this has no evident negative phenotypic consequences from an agronomic perspective. This suggests that the missing pair of chromosome 6As is compensated by the homoeologues from either wheat B- and D- or the *Thinopyrum* genome. The wheat chromosome arm 6AS is known to host the locus Gli-2 responsible for encoding α- and β-gliadins [38], hence potentially influencing the baking qualities of the genotype by altering the gliadin/glutenin ratio. Partial *T. aestivum* × *Thinopyrum* spp. amphiploids CS14PHR21 and 11955 present a consistent karyotype across different individuals, suggesting that their particular genomic constitutions are stable across generations.

Breeding line 202 included plants with variable chromosome numbers (2n = 55–58) and compositions (13–16 *Thinopyrum* and 41–42 wheat chromosomes). Based on chromosome painting, the composition of *Thinopyrum* spp. chromosomes varies among individuals of line 202 and is different from the two other perennial lines. The two types of chromosomes partially painted with pTe probe with specific (GAA)n and pAs1 bands were not observed in any decaploid *Thinopyrum* accession analyzed. The phenotypic segregation within the line 202 including a response to stripe rust infection corresponds to the variability in chromosome constitution observed among its individuals. In spite of its vigorous regrowth, line 202 had the lowest grain yield, i.e., a low fertility, which can be caused by the meiotic abnormalities of its unbalanced karyotype. This line may need further selection for a stabilized chromosome composition. However, all three partial amphiploids analyzed in our work had individuals with 2n = 56, which may be sufficient for transferring substantial post-harvest regrowth [16], as the variability in chromosome composition can influence their performance.

The cytological analysis of potential wild parents allowed for the interpretation of the karyotypes of partial wheat × *Thinopyrum* amphiploids. All three *Thinopyrum* accessions used in our study were decaploids. In the U.S. National Plant Germplasm System, two of them are designated as *Th. elongatum* and one as *Th. ponticum*. The taxonomy of perennial tall wheatgrass is controversial [15,16]. Some researchers designate diploid species of the genera as *Th. elongatum* (Host) D.R. Dewey and those of the decaploid species as *Th. ponticum* (Popd.) Barkworth and D.R. Dewey. Several cytogenetic and molecular phylogenetic studies have demonstrated the allopolyploid nature of decaploid *Th. elongatum* / *Th. ponticum*, though there is no clear consensus regarding its genome formula [15,21]. One of the genome formulas proposed for decaploid *Thinopyrum* is EeEbExStSt [21], reflecting the close relatedness of three subgenomes to a diploid *Thinopyrum* ssp. and the other two other subgenomes to *Pseudoroegneria* ssp. Thus, the difference in distribution of *Th. elongatum*-specific repeats along the chromosomes of potential parental *Thinopyrum* accessions analyzed in our work may reflect their allopolyploidy. Therefore, the partial amphiploids, originating from wheat crosses with a decaploid *Thinopyrum*, may receive different chromosome subsets from the allopolyploid *Thinopyrum* donor. The chromosome numbers reported in U.S. National Plant Germplasm System for PI 206624, PI 547312, and W6 21870 are 70, 68, and 70, respectively. Zheng et. al analyzed the chromosome numbers of *Thinopyrum* accessions from the USDA-ARS collection and found that some samples represented mixed populations with different ploidy levels [39]. We observed variability in the number of chromosomes within two accessions, and the chromosomes of all the accessions had varying distributions of pAs1 and (GAA)n repeats and pTe painting. This may reflect the cross-pollinating nature of the *Thinopyrum* ssp. Accordingly, the partial wheat × *Thinopyrum* amphiploids analyzed in our work may include different subsets of chromosomes, even if they originated from the same cross with a wild perennial allopolyploid parent. The diversity in chromosome composition observed among the three partial amphiploids, CS14PHR21, 11955, and 202, indicates that their crossing may result in unstable karyotypes and reduced fertility. The mixed chromosome composition (i.e., their origin from more than one alien sub-genome) and the rearrangements of wheat chromosomes in meiotically stable and fertile partial amphiploids originating from wheat–*Thinopyrum* crosses has been previously described [22,25,26]. Fedak and Han analyzed hybrids between fertile partial amphiploids and found meiotic abnormalities and reduced fertility. The characterization of breeding lines using FISH could aid in the selection process by ensuring the preservation of the genomes’ integrity, reducing the likelihood of a reduction in the plants’ fertility [25,26,40].

The development of *T. aestivum* × *Thinopyrum* spp. amphiploids that exhibit mechanisms of functional dormancy represents the next crucial breeding target in the efforts to develop a perennial grain crop. The plants will need to successfully transit to vegetative development after having completed their reproductive phase, as observed in perennial grasses from temperate areas. This is a key trait in the differentiation between annual and perennial life histories, manifested as the transition from a monocarpic strategy to a polycarpic one. The genetic underpinnings responsible for a polycarpic strategy in plants have not been completely elucidated [41]; however, work on the polycarpic species *Arabis alpina* L. has contributed to this goal. A single gene named PERPETUAL FLOWERING 1 (PEP1) has been found to be responsible for the transition from reproductive to vegetative development by limiting the length of the flowering phase and inhibiting the development of floral structures on lateral shoots [42]. The breeding lines described in this study are polycarpic but do not revert to vegetative development, instead persisting in the continuous production of new reproductive tillers. This indicates that the *Thinopyrum* spp. chromosomes are sufficient to override the monocarpic strategy expressed by the wheat genome but not to enact an effective switch from reproductive to vegetative development. This could be due to the loss of the *Thinopyrum*-derived genes responsible for producing an appropriate response to environmental signaling, for example, day length sensitivity, or to a regulatory conflict between the common wheat and *Thinopyrum* genomes resulting in day-length-neutral plants. The first hypothesis might be addressed by developing new amphiploids which increase the proportion of *Thinopyrum* chromosomes relative to wheat chromosomes using tetraploid wheat. This is analogous to the development of hexaploid triticale (×*Triticosecale* Wittm. ex A.Camus 2n = 6x = 42. AABBRR) [43]. In this instance, if a diploid *Thinopyrum* species was used, a complete E genome could be added to tetraploid wheat to form a hexaploid with the genomic constitution AABBEE. Alternatively, by leveraging the photoperiod sensitivity observed in certain common wheat cultivars, for example hard red winter wheat cv. “Cheyenne” [44], a new amphiploid could be developed in which the regrowth after harvest becomes more responsive to the photoperiod. In this way, a regulatory framework could be provided to make the amphiploids respond to day-length and hence develop genotypes that will successfully and appropriately cross the boundaries between reproductive and vegetative development.

Ultimately, the ability to inter-breed from primary amphiploids is critical to the progress of developing new types of perennial cereals. However, substantial progress will be made if breeding programs can be facilitated through genetically diverse primary amphiploids, intercrossing them to generate large-scale genetic segregation on which substantial selection pressure can be imposed. The ability to group primary partial amphiploids through the use of FISH may help plant breeders take advantage of the diversity which polyploid donors offer and negate some of the disadvantages of dealing with a synthetic genome. Moreover, the present study offers an example of possible chromosomal rearrangements that can be encountered in the development of agronomically viable perennial wheat breeding lines. The implementation of FISH allowed for the detection of a translocation and the loss of a wheat chromosome pair; instances that could negatively affect the progress of a perennial wheat breeding program by reducing the plant fertility of newly developed hybrids. Based on these observations, the genomic characterization through FISH represents a valid tool to facilitate the establishment of a foundational pool of breeding lines for the development of a perennial wheat crop. Perennial wheat is still under development, and there is ongoing research on its agronomic performance [45]. So far, only one commercial line, Salish Blue originating from *T. aestivum* and *Th. ponticum,* has been released. Recent studies on the field traits and cytology of wheat–*Thinopyrum* amphiploids originating from *Th. Intermedium* [46,47] showed lower grain parameters (grain size, yield, and TKW) than domesticated wheat [46]. In our work, we report the agronomic performance and chromosome constitution of new wheat x *Th. ponticum* partial amphiploids, with good fertility and grain parameters closer to the wheat parent. Perennial grain crops have been and are being attempted by many groups of researchers around the world. The goal is worth the effort.

## 4. Materials and Methods

### 4.1. Germplasm

The breeding lines CS14PHR21, 202, and 11955 were the *T. aestivum* × *Thinopyrum* spp. partial amphiploids used in this study. The first two were developed by the Washington State University Winter Wheat breeding program [48,49], while the latter was kindly provided by the NSW Department of Primary Industries, Cowra, Australia. CS14PHR21 and 202 were derived from a common wheat cv. Chinese Spring/ *Thinopyrum* spp. hybridization which was then backcrossed to common wheat cv. Madsen. The exact pedigree of 11955 is not known but can be described as *T. aestivum*/*Th. ponticum* [50]. The annual hard red winter wheat cultivar ‘CS14PVT03’ was used as a comparison for agronomic performance, given its adaptation to Western Washington State conditions.

To interpret the chromosome composition of *T. aestivum* × *Thinopyrum* spp. amphiploids, we analyzed the karyotypes of potential *Thinopyrum* parents. *Th. elongatum* accessions PI 206624 and W6 21870 along with *Th. ponticum* accession PI 547312 were obtained from the USDA-ARS National Plant Germplasm collection.

### 4.2. Assessment of Agronomic Performance

The amphiploid breeding lines 11955, CS14PHR21, and a common wheat control were planted in 1.2 × 3 m (7 rows, 15 cm apart) plots with three replicates in Fall 2020. The amphiploid breeding line 202 was planted as single plot. All the entries were located on certified organic land in Mount Vernon, WA, USA, and sown to achieve a plant population of 400 plants/m^2^. The plots were fertilized with chicken manure (8-2-4, N-P-K), providing 67 Kg/ha of nitrogen in March 2021. The plots were harvested in August 2021 using a Wintersteiger plot combine. The second-year harvest was completed by hand in August 2022. The PSCR was evaluated starting in September 2021, differentiating between annual (i.e., no regrowth) and regrowing genotypes. The plots were visually scored by the same person using a 0 to 10 scale corresponding to the percentage of the original plant stand still growing (0 = no regrowth to 10 = 100% of the original population returning). The plants were mowed to a height of 15 cm with a self-propelled mower in November 2021. The second-year regrowth was evaluated in November 2021 and May 2022 using the same method described above. The BBCH phenological scale [51] was used to characterize the specific stages of growth observed in the second year. Stripe rust infection was rated based on the plant reaction (resistant, moderately resistant, moderately susceptible, and susceptible) and severity (percentage of leaf tissue affected by the pathogen) according to conventional scoring methods [52]. Weather data was obtained from the WSU AgWeatherNet’s station located in Mount Vernon, WA, USA (+48.44, −122.39) [53].

### 4.3. Kernel Characterization

The thousand-kernel weight was measured by weighing three seed samples of 100 random kernels for each breeding line from each plot and multiplying the weight by ten. The seed length of a sample consisting of 30 random kernels for each breeding line was measured using a digital caliper. The length was measured from the germ to the brush end of each kernel, and the width measured from the left to right side of each kernel.

### 4.4. Statistical Analysis

A one-way analysis of variance was conducted with R (Rstudio 2023.06.1) using “breeding line ID” as the independent factor and an alpha threshold of 0.05 to determine statistical significance. Variance homogeneity was assessed using Levene’s test. Tukey’s honest significant difference post-hoc test was used to establish differences between “breeding line ID”. The “regrowth rate” and “second-year grain yield” data deviated from the normal distribution due to the zero values associated with the plots that did not regrow. In those cases, a non-parametric Kruskal–Wallis test was used to assess the significance of the observed values.

### 4.5. Cytogenetic Analysis

For chromosome preparations and FISH, previously described methods [54,55] with some modifications [56,57] were used. The FISH probe mixture, comprising 10 μL in 2 × SSC-1 × TE buffer, included 150 ng of the E-genome-specific probe pTe (Danilova et al., unpublished) labeled with fluorescein-12-dUTP (PerkinElmer, Waltham, MA, USA), 200 ng of the D-genome repeat-specific probe pAs1 [58,59], containing a mixture of oligonucleotides 5′TEX615-TCAGAGTTCATTTGAAATGCTTTCA and 5′TEX615-AAGTTACAACCTTTTCAGAGTTCATTTGA, 5 ng of oligonucleotide 5′Cy5-(GAA)9 (synthesized by Integrated DNA Technologies, Inc., Coralville, IA, USA), and 750 ng of autoclaved salmon sperm DNA. The pTe probe was produced through PCR amplification of dispersed repeats from diploid *Th. elongatum*. The PCR products were labelled by nick translation according to Kato et al. [55]. DNA denaturation, hybridization, slide washing, and counterstaining were carried out as described by Zhang et al. [57]. The slides were observed using a Zeiss AxioImager M2 microscope, and images were captured using a Zeiss Axiocam 506 and Zeiss AxioVision SE64 Rel. 4.9.1 software, followed by processing with Adobe Photoshop (Adobe Systems Incorporated, San Jose, CA, USA). The combination of pTe, pAs1, and (GAA)n probes in multicolor FISH allowed for the identification of wheat and alien chromosomes, facilitating the identification of individual wheat chromosomes. Chromosome counts and karyotype analyses were performed on 4–7 individuals of every line and 3–9 metaphase spreads of each individual.

## Figures and Tables

**Table 1:** Regrowth rate.

ID		September 2021 (%)	November 2021 (%)	May 2022 (%)
CS14PHR21		93.33 ^a^	86.67 ^a^	17.32 ^a^
202		100 ^1^	100 ^1^	30 ^1^
11955		96.67 ^a^	93.33 ^a^	0 ^a^
CS14PVT03		0 ^b^	0 ^2^	0 ^2^
	St. error mean	15.9	4.47	5
	*p*-value	<0.001	0.51	0.37

^1^ Single plot evaluation. ^2^ Annual growth habit. Values followed by the same superscript are not significantly different.

**Table 2:** Stripe rust infection.

ID		Plant Reaction	Severity (%)
CS14PHR21		MR	26.67 ^a^
202		MR	30 ± 10 ^1^
11955		MS	53.33 ^b^
CS14PVT03		MR	20 ^a^
	St. error mean		5.27
	*p*-value		<0.001

^1^ Severity value is expressed as a range due to the phenotypic segregation observed within the plot. MR = moderately resistant; MS = moderately susceptible. Values followed by the same superscript are not significantly different.

**Table 3:** Kernel characteristics.

ID		Thousand-Kernel Weight (g)	Kernel Length (mm)	Kernel Width (mm)
CS14PHR21		31.79 ^a^	6.34 ^bc^	2.68 ^b^
202		33.1 ^a^	6.17 ^b^	2.53 ^c^
11955		36.98 ^b^	6.93 ^a^	2.67 ^b^
CS14PVT03		45.31 ^c^	5.91 ^c^	3.51 ^a^
	St. error mean	1.03	0.05	0.04
	*p*-value	<0.001	<0.001	<0.001

Values followed by the same superscript are not significantly different.

**Table 4:** Grain yield.

ID		1st Year Grain Yield (Kg/ha)	2nd Year Grain Yield (Kg/ha)
CS14PHR21		1187.41 ^a^	112.96 ^a^
202		661.11 ^1^	283.33 ^1^
11955		1824.24 ^ab^	0 ^a^
CS14PVT03		2367.13 ^b^	0 ^2^
	St. error mean	199.72	73.91
	*p*-value	0.037	0.37

^1^ Breeding line evaluated as single plot. ^2^ Annual growth habit. Values followed by the same superscript are not significantly different.

## Data Availability

Full data set available at breadlab.wsu.edu.

## Supplementary Materials

The following supporting information can be downloaded at: https://www.mdpi.com/article/10.3390/plants12183217/s1, Figure S1: FISH of mitotic chromosomes of decaploid *Thinopyrum* spp. accessions.
