# Supplementary material for: Agronomic Evaluation and Molecular Cytogenetic Characterization of Triticum aestivum × Thinopyrum spp. Derivative Breeding Lines Presenting Perennial Growth Habits
Source: Plants (Basel). 2023 Sep 9;12(18):3217. doi: 10.3390/plants12183217 (PMC10534903; doi:10.3390/plants12183217)
Supplement: Supplementary file 1 [file plants-12-03217-s001.zip › Supplementary figure_S1.pdf]

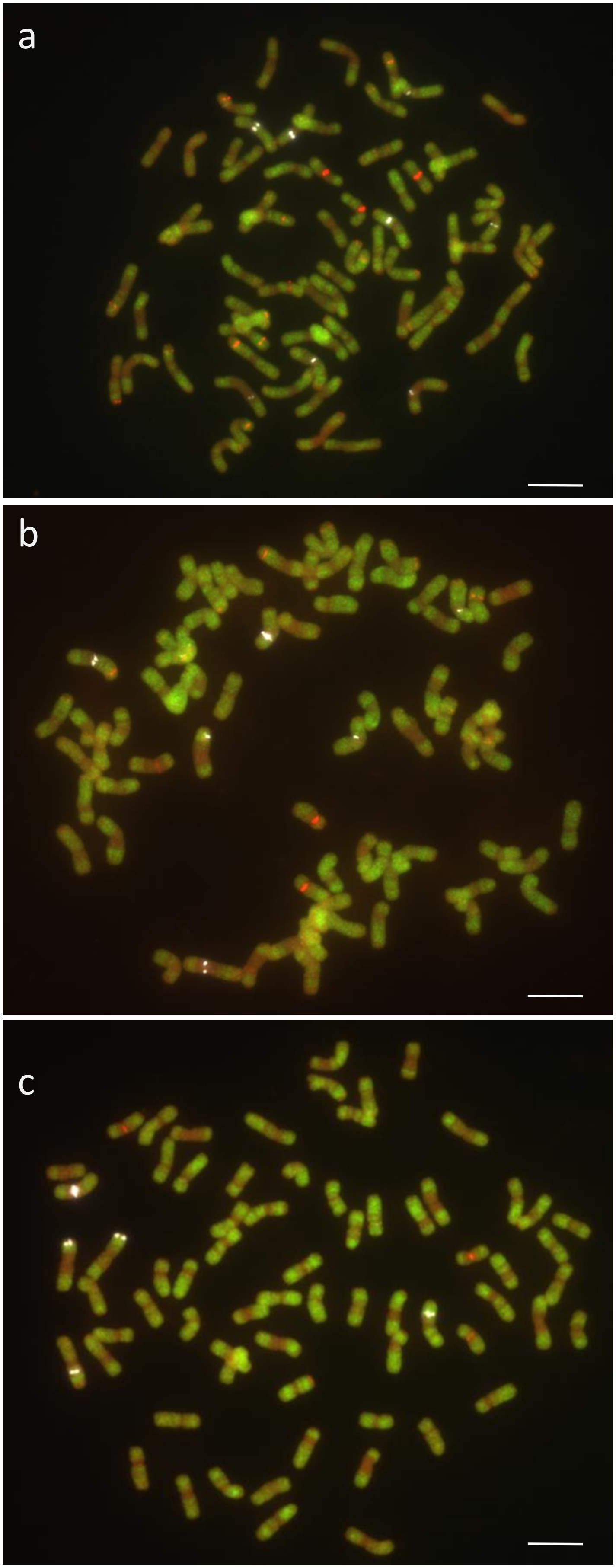

Supplementary Figure 1S: FISH of mitotic chromosomes of decaploid *Thinopyrum sp.* accessions. a) *Th. elongatum* PI 206624, 2n=70, b) *Th. elongatum* W6 21870, 2n=70 c) *Th. ponticum* PI 547312 2n=69. Probes pTe, (GAA)n and pAs1 are shown in green, white, and red, respectively. Chromosomes counterstained with DAPI are shown in dark orange pseudocolor. Bar represents 10 μm.
